# Changes in urinary electrolytes during acute respiratory acid-base modifications

**DOI:** 10.1186/cc13620

**Published:** 2014-03-17

**Authors:** M Ferrari

**Affiliations:** 1 Fondazione IRCCS Ca Granda - Ospedale Maggiore Policlinico, Milan, Italy

## Introduction

The renal system compensates respiratory disorders of acid-base equilibrium by modifying the urinary electrolyte composition [1]. Such a condition mainly involves changes in urinary ammonium excretion as a reaction to prolonged periods of acid-base disequilibrium, leaving the renal response to acute derangements unexplored. We aimed to determine the acute variations of urinary ammonium [NH4+]_u_, sodium [Na+]_u_, potassium [K+]_u _and chloride [Cl]_u _concentrations following controlled minimal respiratory acid- base imbalances, and to further investigate whether urinary anion gap ([AG]_u _= [Na+]_u _+ [K+]_u _- [Cl]_u_) and sodium and chloride difference ([Na+]_u _- [Cl]_u_) might unveil an early activation of the renal response.

## Methods

Patients admitted to the ICU after major surgery were enrolled, during intubation and sedation. Patients with chronic renal failure were excluded. A urinary catheter was connected to the quasi- continuous urinary analyzer KING^®^, measuring [NH4+]_u_, [Na+]_u_, [K+]_u _and [Cl]_u _over time. Based upon the arterial pH (pHa) at entrance, patients were randomly assigned to controlled hypoventilation (30% reduction of minute ventilation if pHa ≥7.40) or hyperventilation (30% increase of minute ventilation if pHa <7.40) for 2 hours. Samples for blood gas analysis were collected every 30 minutes.

## Results

Thirty patients were enrolled; 20 were hypoventilated, 10 hyperventilated. At 2 hours from ventilation change, pHa was respectively decreased from 7.44 ± 0.02 to 7.34 ± 0.02 and increased from 7.37 ± 0.03 to 7.44 ± 0.02 (*P *< 0.001) in the two groups. Mean [NH4+]_u _rose by 2.6 ± 3.3 mEq/l in hypoventilated patients and fell by 2.5 ± 2.4 mEq/l in the hyperventilated (*P *< 0.001). No difference in mean [K+]_u _was observed at any time, while [Na+]_u _and [Cl]_u _progressively decreased in both groups (*P *< 0.05). [Na+]_u _was reduced by 48 ± 43 mEq/l during hypoventilation and by 32 ± 41 mEq/l during hyperventilation (*P *= 0.14), while [Clļ_u _decreased by 29 ± 69 and by 46 ± 66 mEq/l respectively (*P *= 0.93). Whereas [AG]_u _did not differ between groups, [Na+]_u _- [Cl]_u _variation of hyperventilated patients was greater than that of hypoventilated (17 ± 34 vs. -18 ± 54 mEq/l, *P *< 0.05).

## Conclusion

During acute respiratory modifications, changes in urinary ammonium can be observed within the first 2 hours even while maintaining arterial pH within a physiologic range. This response seems to be better associated with changes in the difference between urinary sodium and chloride rather than anion gap.
